# A Novel Piperazine-Based Drug Lead for Cryptosporidiosis from the Medicines for Malaria Venture Open-Access Malaria Box

**DOI:** 10.1128/AAC.01505-17

**Published:** 2018-03-27

**Authors:** R. S. Jumani, K. Bessoff, M. S. Love, P. Miller, E. E. Stebbins, J. E. Teixeira, M. A. Campbell, M. J. Meyers, J. A. Zambriski, V. Nunez, A. K. Woods, C. W. McNamara, C. D. Huston

**Affiliations:** a Department of Medicine, University of Vermont Larner College of Medicine, Burlington, Vermont, USA; b Cellular, Molecular and Biomedical Sciences Graduate Program, University of Vermont, Burlington, Vermont, USA; c California Institute for Biomedical Research, La Jolla, California, USA; d Saint Louis University School of Medicine, St. Louis, Missouri, USA; e Paul G. Allen School for Global Animal Health, College of Veterinary Medicine, Washington State University, Pullman, Washington, USA

**Keywords:** AIDS, cryptosporidiosis, Cryptosporidium, diarrhea, piperazine

## Abstract

Cryptosporidiosis causes life-threatening diarrhea in children under the age of 5 years and prolonged diarrhea in immunodeficient people, especially AIDS patients. The standard of care, nitazoxanide, is modestly effective in children and ineffective in immunocompromised individuals. In addition to the need for new drugs, better knowledge of drug properties that drive *in vivo* efficacy is needed to facilitate drug development. We report the identification of a piperazine-based lead compound for Cryptosporidium drug development, MMV665917, and a new pharmacodynamic method used for its characterization. The identification of MMV665917 from the Medicines for Malaria Venture Malaria Box was followed by dose-response studies, *in vitro* toxicity studies, and structure-activity relationship studies using commercial analogues. The potency of this compound against Cryptosporidium parvum Iowa and field isolates was comparable to that against Cryptosporidium hominis. Furthermore, unlike nitazoxanide, clofazimine, and paromomycin, MMV665917 appeared to be curative in a NOD SCID gamma mouse model of chronic cryptosporidiosis. MMV665917 was also efficacious in a gamma interferon knockout mouse model of acute cryptosporidiosis. To determine if efficacy in this mouse model of chronic infection might relate to whether compounds are parasiticidal or parasitistatic for C. parvum, we developed a novel *in vitro* parasite persistence assay. This assay suggested that MMV665917 was parasiticidal, unlike nitazoxanide, clofazimine, and paromomycin. The assay also enabled determination of the concentration of the compound required to maximize the rate of parasite elimination. This time-kill assay can be used to prioritize early-stage Cryptosporidium drug leads and may aid in planning *in vivo* efficacy experiments. Collectively, these results identify MMV665917 as a promising lead and establish a new method for characterizing potential anticryptosporidial agents.

## INTRODUCTION

Cryptosporidiosis, caused by infection of the gastrointestinal epithelium by Cryptosporidium parasites, is a major cause of life-threatening diarrhea in children, particularly those under the age of 1 year (1–3). It is also highly associated with growth stunting and developmental delays (1, 4–6). Two species, Cryptosporidium hominis and Cryptosporidium parvum, cause >98% of human cases (7). While cryptosporidiosis predominantly affects children in developing countries, it is also the most important cause of waterborne diarrhea in the United States (8) and a frequent cause of diarrhea in immunocompromised individuals, especially AIDS patients and transplant recipients, among whom the infection is typically prolonged and can be fatal (9, 10).

Better treatments for cryptosporidiosis are badly needed. Nitazoxanide, the current standard of care, accelerates recovery in immunocompetent individuals (11). However, nitazoxanide is only partially effective in children and is no better than a placebo in AIDS patients (12, 13). Paromomycin, which is used as a positive control in rodent drug efficacy studies, is also ineffective in AIDS patients (13). Unfortunately, the reasons for nitazoxanide and paromomycin failure are not known. One possibility is that both drugs inhibit Cryptosporidium growth but do not actually kill Cryptosporidium parasites (i.e., they may be parasitistatic rather than parasiticidal), depending on the host's immune system to clear the infection.

Several recent target- and phenotype-based screening efforts have resulted in the identification of multiple lead compounds with promising *in vivo* efficacy (14–24), but there is no established pathway for the development of effective Cryptosporidium drugs (25). Both the lack of a reliably efficacious drug to serve as a benchmark and the variable outcomes of existing leads in different animal models complicate the prioritization of compounds for further development, since the meaning of variable outcomes in different animal models and the compound characteristics that predict efficacy are unknown. Thus, appropriate means of prioritization of such compounds for further development are poorly defined, and new prioritization methods are needed.

Here we report the discovery of a promising new piperazine-based drug lead for the treatment of cryptosporidiosis by use of an immunocompromised mouse model of prolonged infection in combination with a novel *in vitro* assay that is analogous to a classical bacterial time-kill curve assay. By reanalyzing our prior Medicines for Malaria Venture (MMV) Malaria Box screening data (21), we identified MMV665917 as a highly selective Cryptosporidium inhibitor with activity against multiple parasite isolates. Nitazoxanide, clofazimine, and paromomycin were not curative in chronically infected NOD SCID gamma (NSG) mice, but clofazimine and paromomycin were effective in a mouse model of acute infection. On the other hand, MMV665917 was effective in mouse models of both chronic and acute cryptosporidiosis. Measurement of the rate of parasite elimination following exposure to different drug concentrations enabled determination of the concentration of MMV665917 needed to maximize the rate of parasite elimination. Furthermore, the data suggested that MMV665917 was parasiticidal against Cryptosporidium spp., while nitazoxanide, clofazimine, and paromomycin appeared to be parasitistatic. We believe that this parasite persistence assay has general value for Cryptosporidium drug development, since information from it may be useful for prioritizing early-stage drug leads and for planning *in vivo* efficacy studies and understanding their results.

## RESULTS

### Reanalysis of the MMV Malaria Box C. parvum screen identified new inhibitors.

The results from the recently screened MMV Open Access Malaria Box (21) were reanalyzed using a modified hit definition. The mean of parasite numbers normalized to host nucleus numbers was determined for the full library and was set to zero. The results for each compound were then expressed as the distances from the mean and were used to generate a frequency distribution plot, giving rise to a normal distribution (see Fig. S1 in the supplemental material). By use of the 95th percentile as the cutoff, 20 potential inhibitors were identified. Three of the 20 compounds also affected host nucleus numbers and were therefore excluded from further analysis. Fifteen of the remaining 17 compounds were purchased and were confirmed as selective *in vitro* inhibitors of C. parvum development. This reanalysis gave an overall hit rate of 3.75% (15/400) and yielded six Cryptosporidium inhibitors that were not identified in our previous study (see Table S1 in the supplemental material) (21). The parent compounds and/or commercially available variants for 8 of the 15 Cryptosporidium inhibitors were subsequently tested in an immunocompromised mouse model of chronic cryptosporidiosis (see Table S2 in the supplemental material). Only MMV665917 was efficacious at the dose tested.

### MMV665917 is a highly selective Cryptosporidium inhibitor.

**FIG 1:**
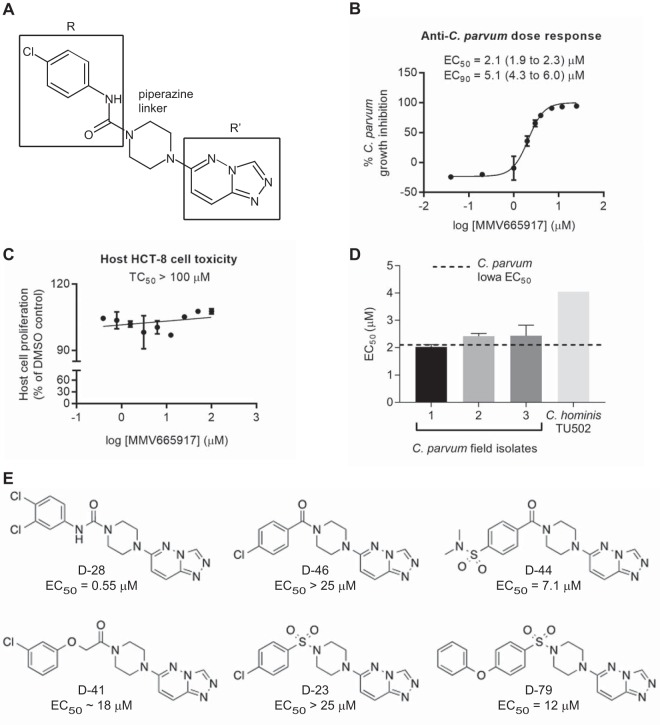
The piperazine-based Malaria Box compound MMV665917 is a selective inhibitor of Cryptosporidium growth *in vitro*. (A) Structure of MMV665917 showing a piperazine linker connecting the indicated R and R′ groups. (B) Dose-response curve showing inhibition of C. parvum growth inside HCT-8 cells after 48 h of incubation. Parasite numbers were normalized to those with DMSO, the vehicle control. Each data point represents the mean from 3 biological replicates, with 4 technical replicates per experiment. Error bars indicate standard deviations. (C) Effect of MMV665917 on the proliferation of host HCT-8 cells as assessed by a CellTiter AQ_ueous_ assay (Promega). Data are means and standard deviations combined from 2 biological replicates with 4 technical replicates each. (D) MMV665917 activity against C. hominis TU502 and three different bovine field isolates of C. parvum. Means and standard deviations from 2 independent experiments, with 4 technical replicates per experiment, are shown, except for C. hominis TU502, where the mean from 3 replicates of 1 biological experiment is shown. (E) Preliminary structure-activity relationship studies using commercially available variants on the R group, demonstrating a preference for aryl urea (D-28) over carboxamides (D-46, D-44), oxyacetamides (D-41), and sulfonamides (D-23, D-79) (also Fig. S2 and Table S2 in the supplemental material).

A piperazine-containing scaffold, MMV665917 (Fig. 1A), appeared to be a particularly promising new hit, since its activity was highly selective for Cryptosporidium and erythrocyte-stage Plasmodium species (26). MMV665917 was previously reported to be inactive in numerous biological assays, including assays against 11 species of bacteria (including, Escherichia coli, Klebsiella pneumoniae, Salmonella enterica serovar Typhimurium, Staphylococcus aureus, and Streptococcus suis), 12 protozoa, and 7 helminths (26). Based on previously reported toxicity profiling (26), MMV665917 was not toxic to zebrafish and had a selectivity index (SI) of >20 for C. parvum over five mammalian cell lines. MMV665917 was also known to have modest plasma protein binding (83.3% and 88.8% for mouse and human, respectively), to have a low potential for significant drug-drug interactions based on low inhibition of six (five human and one mouse) cytochrome P450 (CYP) isoforms (1A2, 2C9, 2C19, 2D6, 3A4-M, and 3A4-T) at 10 μM, and to have a kinetic solubility of 18 μM at pH 7.4 (26). The major known liability for development was hERG inhibition, a marker for potential cardiotoxicity, by 58% at 11 μM (26).

The half-maximal effective concentration (EC_50_) of MMV665917 against asexual stages of the C. parvum Iowa isolate was 2.10 μM (Fig. 1B). There was no toxic effect on host HCT-8 cell proliferation at concentrations as high as 100 μM (Fig. 1C), giving an SI of >47. MMV665917 displayed similar EC_50_s against three C. parvum field isolates recovered from calves and an EC_50_ of 4.05 μM against C. hominis (isolate TU502) (Fig. 1D). A preliminary structure-activity relationship (SAR) study using commercially available analogues showed that changes could be made around the piperazine linker to both the R and R′ groups defined in Fig. 1A without losing anti-Cryptosporidium activity (see Table S3 and Fig. S2 and S3 in the supplemental material). The addition of a second chlorine at the meta position of the urea's terminal aryl ring increased potency (Fig. 1E). Compounds with an aryl urea at the R position were generally more active than aryl carboxamides, oxyacetamides, and sulfonamides (Fig. 1E). It is worth noting that compounds with an altered substituent on the terminal aryl ring of the carboxamide or sulfonamide regained some potency (Fig. 1E, D-44 and D79), suggesting options for further improvement of potency. More-detailed preliminary SAR results are shown in Fig. S2 and S3.

### Oral MMV665917 is curative in both chronic and acute mouse models of cryptosporidiosis.

Most prior *in vivo* studies of potential Cryptosporidium treatments have used one of several self-resolving infection models and therefore have focused on the acute phase of infection (16, 19, 27, 28). Others have used interferon gamma (IFN-γ) knockout (KO) mice to model chronic infection (29), but the results from different research groups are variable, ranging from lethal infection in some cases to self-limited infection in others (19, 23, 24, 29). In our hands, infection of IFN-γ KO mice is self-resolving (19). Therefore, to mimic chronic infection in immunocompromised people and to afford the opportunity to assess relapse after treatment, we developed a new immunocompromised mouse model using NOD SCID gamma (NSG) mice. In addition to the severe combined immunodeficiency (SCID) defect, NSG mice lack known sources of IFN-γ, including the monocyte/macrophage lineage and natural killer cells (30). Our intention was to more cheaply mimic a previously reported model using SCID mice treated with an IFN-γ-neutralizing antibody (31). Much as reported in that study, infection of NSG mice was reliably established by 6 days following oral gavage of 4- to 5-week-old NSG mice with ∼10^5^ C. parvum oocysts, and asymptomatic fecal shedding of oocysts continued for >2 months (data not shown).

**FIG 2:**
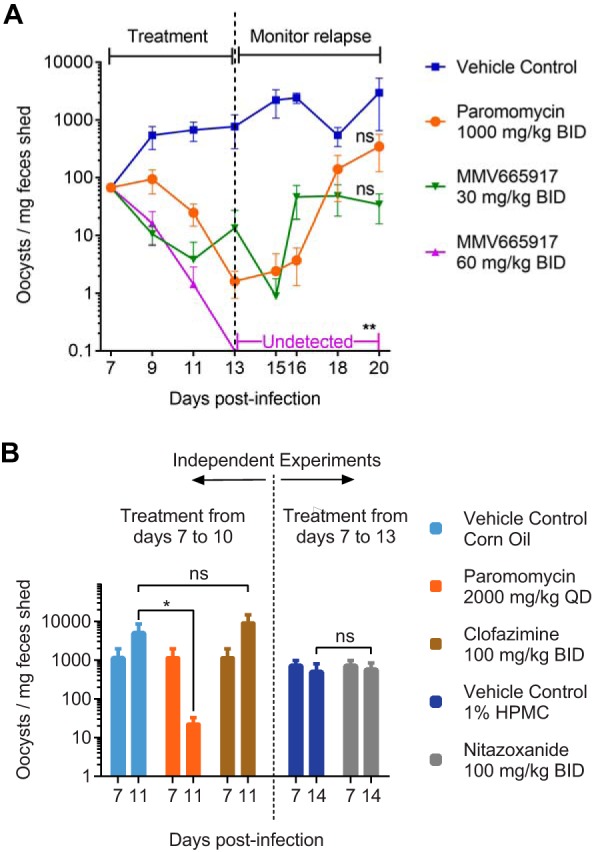
MMV665917 cures NOD SCID gamma mice with established cryptosporidiosis. (A) Efficacy of MMV665917 compared with that of paromomycin (positive control) in NSG mice. NSG mice were infected by oral gavage of C. parvum Iowa isolate oocysts. Fecal parasite shedding was detected by qPCR on day 6, after which animals were treated with the indicated drug regimens for 1 week and were then monitored for relapse of infection. Four mice were used per experimental group, except for paromomycin, for which three mice were used. Data are means and standard errors of the means. Asterisks indicate a significant difference (**, *P* ≤ 0.01), and ns indicates no significant difference (*P* > 0.05), between the data for each treatment and those for the vehicle control by a nonparametric multiple-comparison Kruskal-Wallis test. BID, twice-daily dosing regimen. (B) Short-term efficacies of nitazoxanide, paromomycin, and clofazimine in the NSG mouse model. NSG mice were infected, and fecal parasite shedding was measured by qPCR, as described for panel A. Beginning 7 days after infection, mice were treated with the indicated drug regimens. Fecal parasite shedding was measured just prior to the initiation of treatment and on the day following completion. Four mice were used per experimental group. Data are means and standard errors of the means. The asterisk indicates a significant difference (*P* ≤ 0.05) by the Mann-Whitney test.

Paromomycin (positive control; 1,000 mg/kg of body weight given orally [*per os* {p.o.}] twice daily [BID]) and MMV665917 (30 or 60 mg/kg given orally BID) were compared in the NSG mouse model of cryptosporidiosis, with treatment of mice beginning 7 days after infection. Mice treated with paromomycin for 7 days relapsed promptly upon cessation of treatment (Fig. 2A). MMV665917 given at 30 mg/kg twice daily reduced oocyst shedding by >90%, but as with paromomycin, mice treated at this dose relapsed. Mice treated with MMV665917 at 60 mg/kg twice daily, on the other hand, were apparently cured; no oocyst shedding was observed at any time after the cessation of treatment (Fig. 2A). As with the other commonly used mouse models of cryptosporidiosis, nitazoxanide did not reduce oocyst shedding in NSG mice (Fig. 2B). Clofazimine was also tested using several different vehicles and completely lacked efficacy in the NSG mouse model (Fig. 2B and data not shown [various vehicles]). This was surprising, given its known efficacy in a mouse model of acute infection (19).

**FIG 3:**
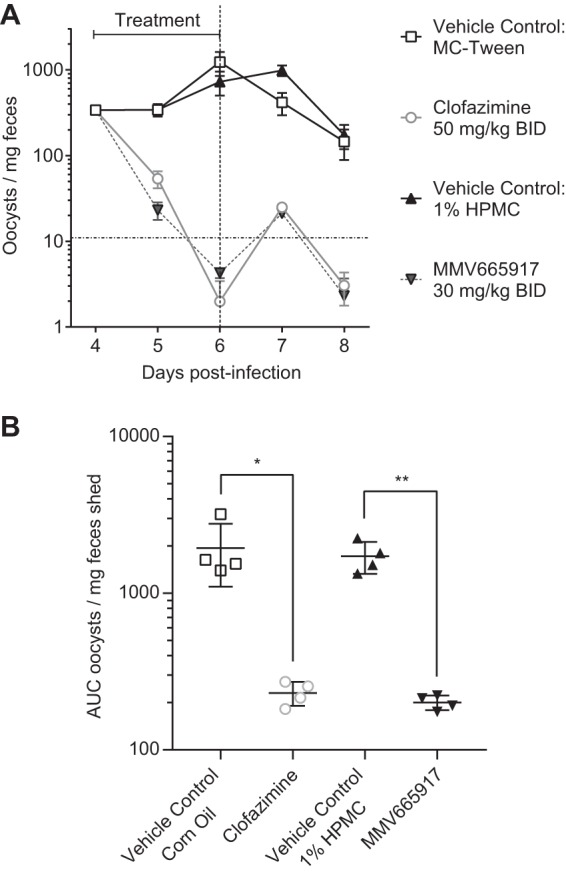
MMV665917 is effective in IFN-γ knockout mice acutely infected with C. parvum. (A) Efficacies of MMV665917 and clofazimine (positive control) in the IFN-γ KO mouse model of acute infection. IFN-γ KO mice were infected by oral gavage of C. parvum Iowa isolate oocysts. Compounds or the indicated vehicles were dosed as indicated twice daily on days 4, 5, and 6 postinfection. Fecal parasite shedding was measured by the isolation of oocysts using sucrose gradient flotation, followed by immunofluorescence staining and detection by flow cytometry. Four mice were used per experimental group. (A) Time course comparing MMV665917 to clofazimine (positive control). Data are means and standard errors of the means. The horizontal dashed line indicates the reliable limit of detection. (B) Total fecal shedding for days 4 to 7. Each symbol represents the result for an individual mouse. GraphPad Prism was used to calculate the area under the fecal shedding-versus-time curve. Data are means and standard deviations. Asterisks indicate significant differences (*, *P* ≤ 0.05; **, *P* ≤ 0.01) by a two-tailed Student *t* test.

To better enable the comparison of results among different mouse models, the efficacy of MMV665917 given at 30 mg/kg twice daily was directly compared with that of clofazimine in an IFN-γ KO mouse model. As noted above, and for unknown reasons, C. parvum infection of IFN-γ KO mice ranges from a self-resolving acute infection to a lethal infection; in our hands, the infection is self-resolving (19). MMV665917 was highly efficacious in this acute-infection model (Fig. 3A and B); the efficacy of clofazimine was also confirmed.

### i.p. dosing of MMV665917.

**FIG 4:**
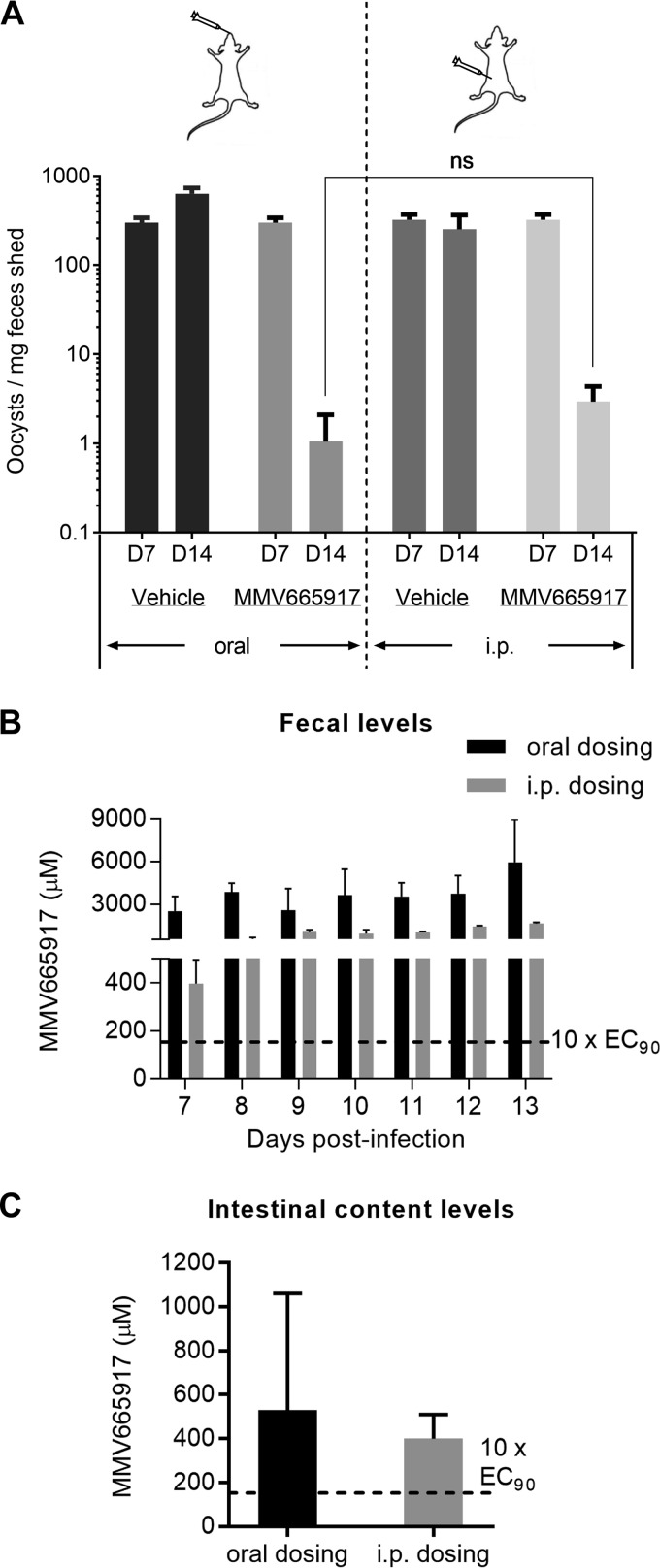
Oral versus intraperitoneal (i.p.) treatment with MMV665917. (A) Efficacy measured by fecal qPCR following the administration of MMV665917 at the indicated dose by either oral gavage or i.p. injection. Efficacy was independent of the dosing route. Data are means and standard errors of the means. Four mice were used for oral dosing and three for i.p. dosing. ns, no significant difference (*P* = 0.2) by a Mann-Whitney test. (B) MMV665917 levels in the feces of mice treated for 7 days orally or i.p. with 60 mg/kg BID from the first 12 h of treatment and every 24 h thereafter. (C) MMV665917 levels in the small intestine following euthanasia 12 to 15 h after the last treatment dose. For panels B and C, data are expressed as micromolar concentrations of MMV665917, calculated by considering 1 g of intestinal contents or feces to be equivalent to 1 ml.

In hopes of determining if MMV665917 efficacy was due to intestinal or oral exposure, we compared the efficacies of oral and intraperitoneal (i.p.) MMV665917 in the NSG mouse model. MMV665917 was equally efficacious regardless of the route of administration (Fig. 4A). This suggested that either (i) MMV665917 undergoes biliary excretion into the intestinal lumen, (ii) systemic compound concentrations drive *in vivo* efficacy of MMV665917, or (iii) both.

Fecal MMV665917 concentrations were determined during treatment of cryptosporidiosis, and fecal MMV665917 levels vastly exceeded the measured EC_90_ regardless of the route of administration (Fig. 4B). In fact, very high levels of MMV665917 were detected in the feces shortly after even a single 60-mg/kg i.p. dose (approximately 78× EC_90_ versus 490× EC_90_ for the corresponding oral dose) (Fig. 4B). Following euthanasia on day 14, between 12 and 15 h after the final dose, the intestinal contents included MMV665917 at many times the EC_90_ regardless of the route of administration (Fig. 4C). Unfortunately, serum samples from these infected NSG mice treated orally or i.p. with MMV665917 were lost during shipping. Based on an independent pharmacokinetic (PK) exposure study in CD-1 male mice, plasma MMV665917 levels continued to increase for 9 h following the administration of a single oral dose of 55 mg/kg, demonstrating that in contrast to the immediately elevated fecal levels observed, plasma MMV665917 concentrations build over time (see Fig. S4 in the supplemental material). The possible interpretation of these data is limited, since the terminal time point was quite early. Collectively, however, these data demonstrated biliary excretion of MMV665917 but failed to provide any insight into whether MMV665917 works via presence in the gut lumen, the tissue, or both.

### Rate of parasite elimination.

**FIG 5:**
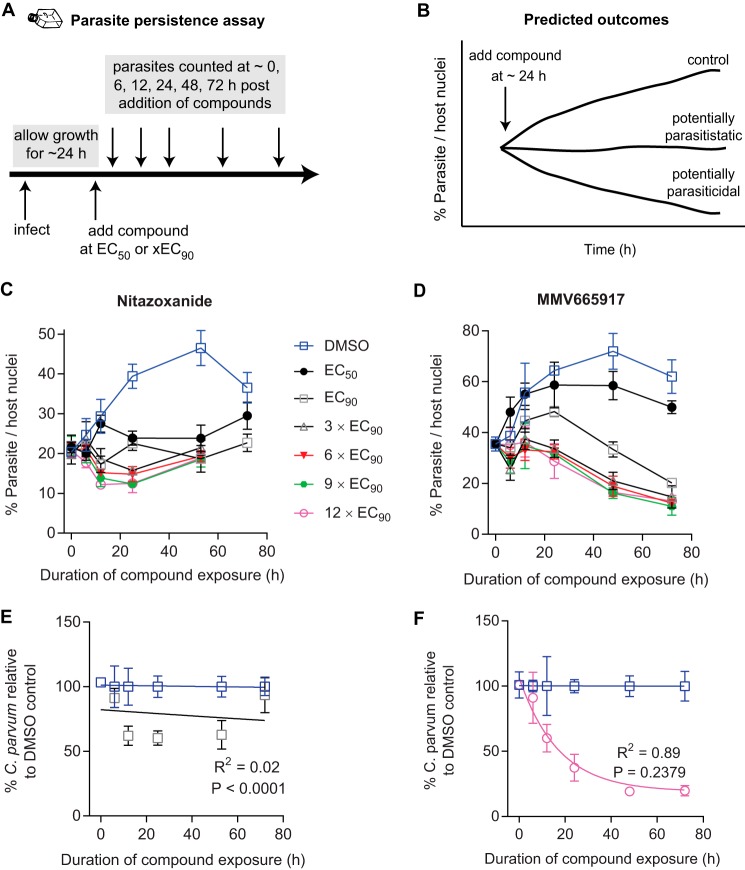
Parasite persistence assay showing *in vitro* elimination of C. parvum following MMV665917 exposure in contrast with parasite persistence in the presence of nitazoxanide. (A) Experimental design of the parasite persistence assay. After the establishment of an infection for approximately 24 h, compounds are added at various concentrations as labeled, and then parasites and host cells are enumerated at multiple time points using immunofluorescence microscopy. (B) Cartoon showing predicted outcomes for potentially parasiticidal or parasitistatic compounds compared to that for the vehicle control (DMSO). (C and D) Parasite numbers normalized to numbers of host nuclei over time with increasing concentrations of nitazoxanide (C) or MMV665917 (D). The data shown are means and standard deviations for 4 culture wells per data point and are representative of 3 independent experiments. (E and F) One-phase exponential decay curve fit for nitazoxanide (E) and MMV665917 (F) using parasite persistence assay data normalized to a percentage of the data for the DMSO control at each time point. Data points are means and standard deviations for 4 culture wells per time point and are representative of 3 independent experiments. The *P* value is the replicates test result (note that a *P* value of ≥0.05 indicates a valid curve fit).

We developed an *in vitro* parasite persistence assay, adapted from classical antibacterial time-kill curves, to determine the concentration of compound required to achieve the maximal anti-Cryptosporidium response, as well as the rates of parasite elimination following exposure to different compound concentrations. A similar approach has been applied by the Medicines for Malaria Venture to compare antimalaria drug candidates with benchmark compounds in immunocompromised mice (32, 33). C. parvum cannot be continuously cultured using simple *in vitro* methods; growth in epithelial monolayers peaks at ∼60 h postinfection. In the parasite persistence assay, we used this narrow time window to mimic the treatment of an established *in vivo* infection by allowing infection of epithelial cell monolayers to progress for 24 h, then adding compounds at the EC_50_ or multiples of the EC_90_, and finally sequentially quantifying parasite numbers (Fig. 5A, schematic). Predicted outcomes include continued growth of control-treated parasites, parasite growth inhibition with potentially parasitistatic (or slowly parasiticidal) compounds, and parasite elimination with rapidly parasiticidal compounds (Fig. 5B). In this assay, parasites persist at the highest nontoxic concentrations of nitazoxanide, paromomycin, and clofazimine (Fig. 5C; see also Fig. S5 in the supplemental material). MMV665917, on the other hand, reduced parasite numbers at concentrations higher than the EC_90_, with a maximal rate of parasite elimination achieved at 3× EC_90_ (Fig. 5D). However, significant parasite numbers remained at 72 h following even the highest-dose treatment with MMV665917. To further assess if MMV665917 results in parasite elimination compared to paromomycin, an extended-treatment experiment was performed in which the culture medium and drugs were replaced every 3 days for a total of 14 days (i.e., 13 days of drug exposure) (see Fig. S6 in the supplemental material). As expected, parasite numbers continued to fall during treatment with the vehicle alone, reaching a low of ∼6% of host cells infected by day 13. Paromomycin treatment results closely mirrored the pattern for the vehicle control, dimethyl sulfoxide (DMSO), at 5 days of culture and beyond, but MMV665917 treatment resulted in a progressive decline in the percentage of host cells infected, to nearly zero by day 13 of treatment (∼0.1% of host cells positive). These data suggested that MMV665917 is parasiticidal for C. parvum, while paromomycin is parasitistatic.

Data from the parasite persistence assay were next used to estimate the *in vitro* rate of parasite elimination in the presence of MMV665917, nitazoxanide, paromomycin, or clofazimine. The parasite numbers for the highest nontoxic concentration tested were expressed as a percentage of those with the DMSO control for each time point in order to isolate the anti-Cryptosporidium effect attributable to each drug, and one-phase exponential decay curves were fit to calculate decay constants (Fig. 5E and F; also Fig. S5 in the supplemental material). Note that it was not possible to fit exponential decay curves to the data for nitazoxanide, paromomycin, or clofazimine (*P*, <0.05 for each by the replicates test [GraphPad Prism]), but a high-quality curve was readily generated for MMV665917. The maximal rate of parasite elimination was achieved at 3× EC_90_ of MMV665917, and the rates of parasite decay were similar for 3× EC_90_ and 12× EC_90_ (decay rate constants [*r*], 0.05346 and 0.05864 for 3× EC_90_ and 12× EC_90_, respectively). The difference in the ability to fit decay curves for MMV665917, nitazoxanide, clofazimine, and paromomycin gave an objective indication that MMV665917 reduced parasite numbers under the conditions of the parasite persistence assay in a manner distinct from the other drugs tested.

## DISCUSSION

The most important result of this study is the identification of MMV665917 as a novel, piperazine-based lead compound for the treatment of cryptosporidiosis. It is active against C. hominis and field isolates of C. parvum, shows no *in vitro* cytotoxicity at high concentrations, and, in agreement with previously published data on the MMV Malaria Box (26), is highly specific for Cryptosporidium parasites and blood-stage Plasmodium species. MMV665917 appears to cure established cryptosporidiosis in highly immunocompromised NSG mice, unlike paromomycin (the most commonly used positive control), nitazoxanide, and the recently identified repurposing lead clofazimine. MMV665917 is also highly efficacious in an IFN-γ KO mouse model of acute infection. Interestingly, MMV665917 is efficacious in NSG mice regardless of dosing by the oral or the i.p. route and accumulates rapidly in the feces following i.p. dosing, indicating that it is at least partially excreted via the biliary tract. To further understand the *in vivo* efficacy of MMV665917, we developed and used a new *in vitro* pharmacodynamic (PD) assay to measure parasite elimination versus time at various concentrations of the compound. Efficacy in the NSG mouse model of chronic infection correlated with progressive parasite elimination in the presence of the compound, since MMV665917 appeared to be parasiticidal and resulted in parasite elimination over time, while nitazoxanide, paromomycin, and clofazimine all appeared to be parasitistatic. These features of MMV665917 and the methods used to define them provide guidance for further development of this piperazine-based compound series to treat cryptosporidiosis. Given the lack of a clearly defined developmental pathway for anticryptosporidials, we believe that these studies provide general guidance for Cryptosporidium drug development.

Since MMV665917 is active against both C. hominis and a variety of C. parvum isolates and is relatively specific for Cryptosporidium (26), it is an especially promising lead. The *in vivo* efficacy studies done here, however, used only C. parvum and nonclinical rodent models of cryptosporidiosis. While both C. parvum and C. hominis cause human disease, and the two species are also believed to be distributed similarly within tissues, it should be noted that the majority of children with life-threatening cryptosporidiosis are actually infected with C. hominis (2). Thus, additional early-stage testing is needed as proof of principle for *in vivo* treatment of C. hominis with MMV665917. The gnotobiotic piglet model is the preferred model for such studies (34). Similarly, MMV665917 needs to be tested in a clinical model of cryptosporidiosis in which the host develops diarrhea, such as the dairy calf model (22, 35, 36).

Although no *in vitro* cytotoxicity was observed with MMV665917 for host cell lines, and it is well tolerated in zebra fish and mice, the inhibition of hERG (58% at 11 μM) presents a potential cardiotoxicity liability. This is confounded by the modest potency of the compound against Cryptosporidium, which suggests the possibility of a narrow therapeutic window in animals (26). The results of the *in vitro* Cryptosporidium time-kill curve assay should aid in the design of animal studies to optimize safety and efficacy, since ignoring protein binding, a concentration equivalent to three times the EC_90_ of the compound maximized the antiparasitic effect. The preliminary SAR studies presented here also suggest that this hERG liability may be addressed through medicinal chemistry to significantly improve compound potency and/or reduce hERG inhibition. Of course, such a program would be aided by identification of the molecular mechanism of action of MMV665917, which remains unknown.

Since MMV665917 was present in the feces regardless of administration via the oral or i.p. route, no conclusion is possible about the PK characteristics that drive *in vivo* efficacy (i.e., intestinal versus systemic exposure). This is a very important area of investigation for Cryptosporidium drug development in general, since the parasite resides in an unusual parasitophorous vacuole on the luminal surface, but enclosed within intestinal epithelial cells. The fecal MMV665917 levels measured provide only an indirect view of cumulative drug exposure in the intestinal mucosa, since these levels include both compound being eliminated and compound that is never absorbed after oral administration. Furthermore, the day-to-day variability seen in Fig. 4B may simply represent variability in drug excretion and/or fecal volumes. The important conclusion from the limited PK analysis performed here is that MMV665917 is at least partially excreted in the feces and presumably undergoes enterohepatic recirculation. Further studies to define the PK characteristics that drive MMV665917 *in vivo* efficacy are needed and should aid in medicinal chemistry and formulation strategies for optimization.

Variability in outcomes in different rodent models of cryptosporidiosis and the lack of knowledge of the basis and significance of such variability greatly complicate Cryptosporidium drug development. Our approach to comparing results in chronically infected NSG mice with results in an IFN-γ KO mouse model of acute cryptosporidiosis suggests that the NSG mouse model of chronic infection sets a more-stringent standard than acute models, since MMV665917 was highly efficacious in both models, but several compounds with good efficacy in an IFN-γ KO mouse model of acute infection (i.e., clofazimine and paromomycin) were ineffective in NSG mice (data not shown). However, it should be stressed that this conclusion is based on only a small number of compounds and that, in any case, it remains unknown if such a high standard is required to achieve reliable efficacy in people. In addition, we have included data for only one model of chronic infection and one of acute infection, and results may differ further among the other available models. The best pathway for developing anti-Cryptosporidium drugs is still being determined, and the predictive value of Cryptosporidium animal models for drug efficacy within different patient populations is not known. Our data suggest that the NSG mouse model of chronic infection is more stringent than the IFN-γ KO mouse model of acute (i.e., self-curing) infection. As noted above, in some mouse facilities, infection of IFN-γ KO mice is lethal (23, 24, 29) and may represent a similarly high stringency model. In any case, there is likely value to prioritizing compounds in development by testing them in animal models of differing stringencies. A lack of efficacy in the NSG model may not preclude high-value compounds from advancing in development (e.g., repurposing of clofazimine); however, this model may help to further differentiate and prioritize the growing number of lead compounds that have been discovered recently. It is logical that efficacy in a mouse model of chronic infection, such as the NSG model, may predict drugs that will be efficacious for the treatment of AIDS patients with chronic cryptosporidiosis and of severely malnourished children.

The data also suggest that efficacy in NSG mice, which lack all aspects of adaptive immunity, may depend on the ability of a compound to eliminate parasites in the absence of a competent immune system (i.e., parasite killing, or parasiticidal activity) and/or the rate of parasite elimination at physiologically relevant compound concentrations. By quantifying parasite persistence versus elimination in the presence of different drug concentrations, it is possible to determine the concentration required to maximize the effect of a compound. In the absence of a simple continuous-culture system, it is not formally possible to prove parasiticidal activity on a routine basis (e.g., use of the recently reported hollow-fiber culture system [37] would require a large amount of compound and be prohibitively expensive). However, this time-kill curve assay enables one to determine whether a compound eliminates parasites or simply blocks further growth during the time frame of the assay, which likely indicates whether a compound is parasiticidal or parasitistatic under the conditions tested. More importantly, this method is simple and inexpensive and requires only a small amount of compound, so it provides the opportunity to directly compare different compounds or compound classes to aid in the prioritization of early-stage drug leads. The number of compounds studied is too small to enable an absolute conclusion, but based on our experience with the NSG mouse model and by analogy with general principles for the treatment of infections in highly immunocompromised individuals, it is likely that parasiticidal compounds will be of the greatest value for the treatment of cryptosporidiosis in the very patients for whom nitazoxanide is either ineffective (e.g., AIDS patients and/or transplant patients) or only modestly effective (e.g., malnourished children). In this regard, our MMV665917 data are in keeping with a recently proposed ideal product profile for an anticryptosporidial drug (25).

In summary, we propose MMV665917 as a promising lead for further development of an anticryptosporidial drug, and we present new methods with general value for Cryptosporidium drug development. Current studies with MMV665917 include testing in a clinical model of infection in dairy calves, additional SAR studies to eliminate hERG inhibition and improve potency, and efforts at drug-target identification.

## MATERIALS AND METHODS

### Reanalysis of MMV Malaria Box screening data.

The screening data published previously for the MMV Malaria Box used a screening “hit” definition of 80% inhibition to identify potential Cryptosporidium growth inhibitors (21). These data were reanalyzed to identify additional Cryptosporidium inhibitors by assuming that the average compound in the collection had no effect and using a statistical approach to identify compounds that differed from the average. For this purpose, the average number of parasites per host nucleus for all compounds was set to zero effect. Data for each compound were then normalized to this average, and the difference from the population mean for each compound was plotted on a frequency distribution plot. Using GraphPad Prism, version 6.01, normality was tested with the D'Agostino-Pearson omnibus K2 test, and the 95th percentile range was calculated. Inhibitors at or beyond the 95th percentile were considered hits.

### Cell culture and parasites.

Human ileocecal adenocarcinoma (HCT-8) cells (ATCC) were cultured in RPMI 1640 medium (Invitrogen) supplemented with 10% heat-inactivated fetal bovine serum (Sigma-Aldrich), 120 U/ml penicillin, and 120 μg/ml streptomycin (ATCC) at 37°C under 5% CO_2_. HCT-8 cells were used between passages 9 and 39 for all experiments. C. parvum Iowa isolate oocysts were purchased from Bunch Grass Farm (Deary, ID). Oocysts were stored in phosphate-buffered saline (PBS) with penicillin and streptomycin at 4°C and were used within 5 months of shedding. C. hominis TU502 isolate oocysts were purchased from the Tzipori laboratory (Tufts University), and C. parvum field isolates were provided by Jennifer Zambriski (Washington State University) and Daryl Nydam (Cornell University).

### Immunofluorescence assay for the determination of Cryptosporidium growth inhibition.

Inhibition of C. parvum growth was measured as described previously (20). Oocysts were excysted by treatment with 10 mM hydrochloric acid (10 min at 37°C), followed by exposure to 2 mM sodium taurocholate (Sigma-Aldrich) in PBS for 10 min at 16°C. Excysted oocysts were then added to >95% confluent HCT-8 cell monolayers in 384-well plates at a concentration of 5,500 Iowa isolate oocysts per well. For C. parvum field isolates, the inoculum required to yield an infection level similar to that produced by 5,500 Iowa isolate oocysts per well was determined and was used for subsequent assays of parasite inhibition. Compounds were added just before or 3 h after infection, and assay plates were incubated for 48 h postinfection at 37°C under 5% CO_2_. Wells were then washed three times with PBS containing 111 mM d-galactose, fixed with 4% paraformaldehyde in PBS for 15 min at room temperature, permeabilized with 0.25% Triton X-100 for 10 min at 37°C, washed three times with PBS with 0.1% Tween 20, and blocked with 4% bovine serum albumin (BSA) in PBS for 2 h at 37°C or 4°C overnight. Parasitophorous vacuoles were stained with 1.33 μg/ml of fluorescein-labeled Vicia villosa lectin (Vector Laboratories) diluted in 1% BSA in PBS with 0.1% Tween 20 for 1 h at 37°C, followed by the addition of Hoechst 33258 (AnaSpec) at a final concentration of 0.09 mM diluted in water for another 15 min at 37°C. Wells were then washed five times with PBS containing 0.1% Tween 20. A Nikon Eclipse TE2000 epifluorescence microscope with an automated stage was programmed using NIS-Elements Advanced Research software (Nikon, USA) to focus on the center of each well and take a 3-by-3 composite image using an EXi Blue fluorescence microscopy camera (QImaging, Canada) with a 20× objective (numerical aperture, 0.45). Nucleus and parasite images were exported separately as .tif files and were analyzed using macros developed on the ImageJ platform (National Institutes of Health) (20). The only modification from the published macro used to count parasites was that the lower size threshold for parasites was decreased from 16.5 to 4 pixels (1 pixel = 0.65 μm). Graphs were plotted, and half-maximal effective concentration (EC_50_) and 90% effective concentration (EC_90_) values were calculated using GraphPad Prism software, version 6.01. For every C. parvum field isolate experiment, a dose-response experiment against the C. parvum Iowa isolate was performed simultaneously as a reference.

Cryptosporidium hominis growth inhibition assays were performed at the California Institute for Biomedical Research (Calibr), San Diego, CA, using a slightly modified immunofluorescence assay to enable automated compound handling and the use of 1,536-well microtiter plates (19). The HCT-8 culture medium was replaced with RPMI 1640 medium supplemented with 2% heat-inactivated horse serum, 100 U/ml penicillin, and 100 mg/ml streptomycin 24 h prior to infection with either C. hominis or the C. parvum Iowa isolate, used as a reference. For these assays, d-galactose was found to be unnecessary and was eliminated from the plate wash buffer. Imaging was performed using a CellInsight CX5 High Content screening platform (Thermo Scientific) with a 10× objective and acquisition of one microscopic field per well. Images were processed using HCS Studio Scan software, and the selected-object count (HCT-8 cells) and spot count (C. hominis) were analyzed in Genedata Screener (version 13.0; Standard). Dose-response curves and EC values were calculated using the Smart Fit function of Genedata Analyzer.

### Parasite persistence assay.

Excysted C. parvum oocysts were added to >90% confluent HCT-8 cells in 384-well plates. Compounds were added at the EC_50_ or multiples of the EC_90_ ∼24 h after infection. At ∼24 h (i.e., the time of compound addition) and at various time intervals thereafter, parasites were washed, fixed, permeabilized, stained, and imaged as for the C. parvum growth assay. For the extended-compound-exposure experiments, the medium and compound were replaced every 3 days. A separate 384-well plate was used for each time interval. Parasite numbers were normalized to host cell nucleus numbers and are expressed as the percentage of parasites per host cell nucleus. In order to fit parasite decay curves, the effect of drugs was isolated from expected changes in parasite numbers over time by expressing the number of parasites as the percentage of the number of parasites with the control, dimethyl sulfoxide (DMSO), for each time point. Exponential decay curves were fit using GraphPad Prism software, version 6.01, and curve validity was assessed using the replicates test.

### Host cell toxicity assay.

Host cell toxicity was measured as described previously (20). HCT-8 cells were grown to >95% confluence in 384-well plates. Increasing concentrations of compounds were added, and assay plates were incubated at 37°C under 5% CO_2_ for 48 h. The corner wells of each plate were trypsinized to remove cells and were used as blanks for measuring absorbance at 490 nm. Cell proliferation was measured using the CellTiter AQ_ueous_ assay kit (Promega, USA) according to the manufacturer's instructions and was expressed as the percentage of proliferation with the vehicle control (DMSO). GraphPad Prism software, version 6.01, was used to plot graphs and calculate the concentration that inhibits 50% of host cell proliferation relative to that with the DMSO control (50% toxic concentration [TC_50_]). A selectivity index (SI) was calculated as the ratio of the C. parvum EC_50_ to the host cell TC_50_.

### PK measurements.

Mouse single-dose plasma pharmacokinetic (PK) studies were performed previously as part of the Malaria Box program (26), and data were kindly provided by the Medicines for Malaria Venture (Geneva, Switzerland). Three overnight-fasted CD-1 male mice were each given an oral suspension of 55 mg/kg of MMV665917 in a 5% DMSO solution in 1% hydroxypropyl methylcellulose (HPMC). Blood samples were collected at 0.083, 0.25, 1, 2, 4, 6, and 9 h posttreatment, transferred to a microcentrifuge containing 1,000 IU/ml of sodium heparin, and spun at 3,000 × *g* for 15 min at 4°C. MMV665917 levels were then measured by liquid chromatography-tandem mass spectrometry (LC–MS-MS) using an API 4000 AB Sciex Instruments mass spectrometer with a Phenomenex Kinetex C_18_ (particle size, 2.6 μm; inside diameter, 2.1 mm; length, 50 mm) column (phase A, 0.1% formic acid–4.9% acetonitrile–95% water; phase B, 0.1% formic acid–4.9% water–95% acetonitrile). The compound spiked into control plasma was used as a standard.

Concentrations of MMV665917 in feces and intestinal contents were measured following compound extraction. Feces or intestinal contents were homogenized in PBS (0.1 g/ml) in a polypropylene tube and were then further diluted prior to the addition of an internal standard (enalapril) and protein precipitation with acetonitrile. The supernatant was then transferred to a fresh polypropylene tube and was dried using a SpeedVac system. Samples were then resuspended and were analyzed using LC–MS-MS.

### *In vivo* efficacy.

All NOD SCID gamma mouse studies were performed in compliance with animal care guidelines and were approved by the University of Vermont Institutional Animal Care and Use Committee. NOD SCID gamma mice with normal flora (NOD.Cg-*Prkdc^scid^ Il2rg^tm1Wjl^*/SzJ) (38) were purchased from The Jackson Laboratory (Bar Harbor, ME, USA) and were housed for at least a week for acclimatization. At the age of 4 to 5 weeks, mice were infected with 10^5^ C. parvum Iowa isolate oocysts. Fecal oocyst shedding is detected 6 days after infection using a quantitative PCR (qPCR assay), so treatment was started on day 7 after infection. Mice (4 per experimental group) were treated either orally (p.o.) with MMV665917 at 30 or 60 mg/kg BID, intraperitoneally (i.p.) with MMV665917 at 60 mg/kg BID, or p.o. with 1,000 mg/kg paromomycin BID. MMV665917 was suspended in DMSO, sonicated three times, for 30 s each time, to obtain a fine suspension, aliquoted, and stored at −80°C for <10 days. On the day of treatment, aliquots of MMV665917 diluted in DMSO were thawed, mixed well using a vortexer, diluted with 1% HPMC, sonicated as before (three times for 30 s each time), mixed well, and given to mice either p.o. or by i.p. injection. A final concentration of 5% DMSO was used in 100 μl of 1% HPMC per dose. For the additional Malaria Box compounds and compound variants tested, the doses were prepared in the same way, and the specific dosages tested are given in Table S3 in the supplemental material. Mice were treated for either 4 or 7 days, allowed to recover for a week, and then sacrificed. Oocyst shedding in feces was monitored throughout by qPCR, including time points while on treatment and while monitoring for relapsed infection following completion of treatment in Fig. 2A.

All interferon gamma (IFN-γ) knockout mouse studies were performed in compliance with animal care guidelines and were approved by the Explora BioLabs (San Diego, CA) Institutional Animal Care and Use Committee. Four-week-old female C57BL/6 IFN-γ^−/−^ mice with normal flora were purchased from The Jackson Laboratory and were acclimated for 3 days prior to infection by oral gavage with 10^6^ C. parvum Iowa isolate oocysts (Sterling Parasitology Laboratory, University of Arizona) suspended in sterile distilled water. At various times postinfection, mice were treated with the compound vehicle alone, clofazimine (used as a positive control), or MMV665917. As described previously, fecal parasite shedding was quantified at various times postinfection by isolating oocysts using a sucrose gradient centrifugation method (39), followed by staining for immunofluorescence microscopy using a fluorescein isothiocyanate-conjugated mouse anti-Cryptosporidium antibody (0.25 μg per sample) and analysis with a Guava easyCyte flow cytometer and CytoSoft data acquisition and analysis software (version 5.3; Guava Technologies, Inc.). Oocyst counts per milliliter of sample were exported to Excel (Microsoft Corp.) and were normalized to counts per milligram of feces. Final data analysis and graphing were carried out using GraphPad Prism software (version 6.01).

## Supplementary Material

Supplemental material
