# Supplementary material for: A Novel Piperazine-Based Drug Lead for Cryptosporidiosis from the Medicines for Malaria Venture Open-Access Malaria Box
Source: Antimicrob Agents Chemother. 2018 Mar 27;62(4):e01505-17. doi: 10.1128/AAC.01505-17 (PMC5913971; doi:10.1128/AAC.01505-17)
Supplement: Supplemental material [file supp_62_4_e01505-17__index.html]

A Novel Piperazine-Based Drug Lead for Cryptosporidiosis from the Medicines for Malaria Venture Open-Access Malaria Box — Supplemental material 

# A Novel Piperazine-Based Drug Lead for Cryptosporidiosis from the Medicines for Malaria Venture Open-Access Malaria Box

## Supplemental material

- Supplemental file 1 -

  Fig. S1 to Fig. S6, Tables S1 to S3

  PDF, 651K
