# Supplementary material for: A Novel Piperazine-Based Drug Lead for Cryptosporidiosis from the Medicines for Malaria Venture Open-Access Malaria Box
Source: Antimicrob Agents Chemother. 2018 Mar 27;62(4):e01505-17. doi: 10.1128/AAC.01505-17 (PMC5913971; doi:10.1128/AAC.01505-17)
Supplement: Supplemental material [file AAC.01505-17_zac004186999s1.pdf]

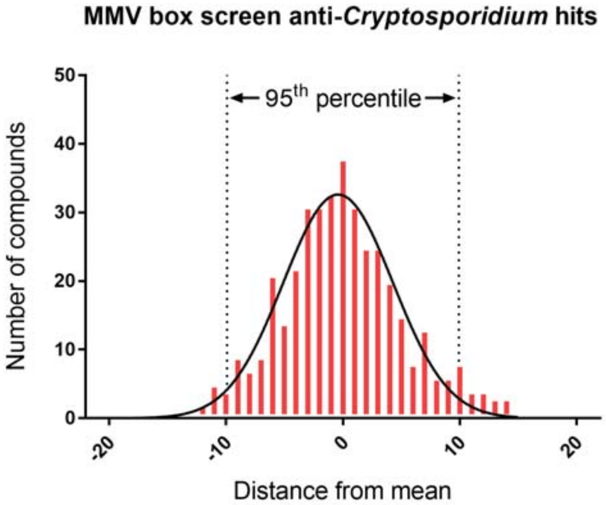

Supplementary Figure 1. Re-analysis of MMV box screening data identifies new *Cryptosporidium* inhibitors. The previously published screening data of 400 compounds of the MMV box were re-analyzed (1). Parasite numbers were normalized to nuclei and expressed as percent nuclei. Mean of % parasite / nuclei was determined and each individual value subtracted from the cumulative mean to determine the distance from mean. A frequency distribution plot of each compound's distance from the mean gave rise to a normal distribution. The upper 95th percentile concentration was set as a cut-off to identify potential inhibitors.

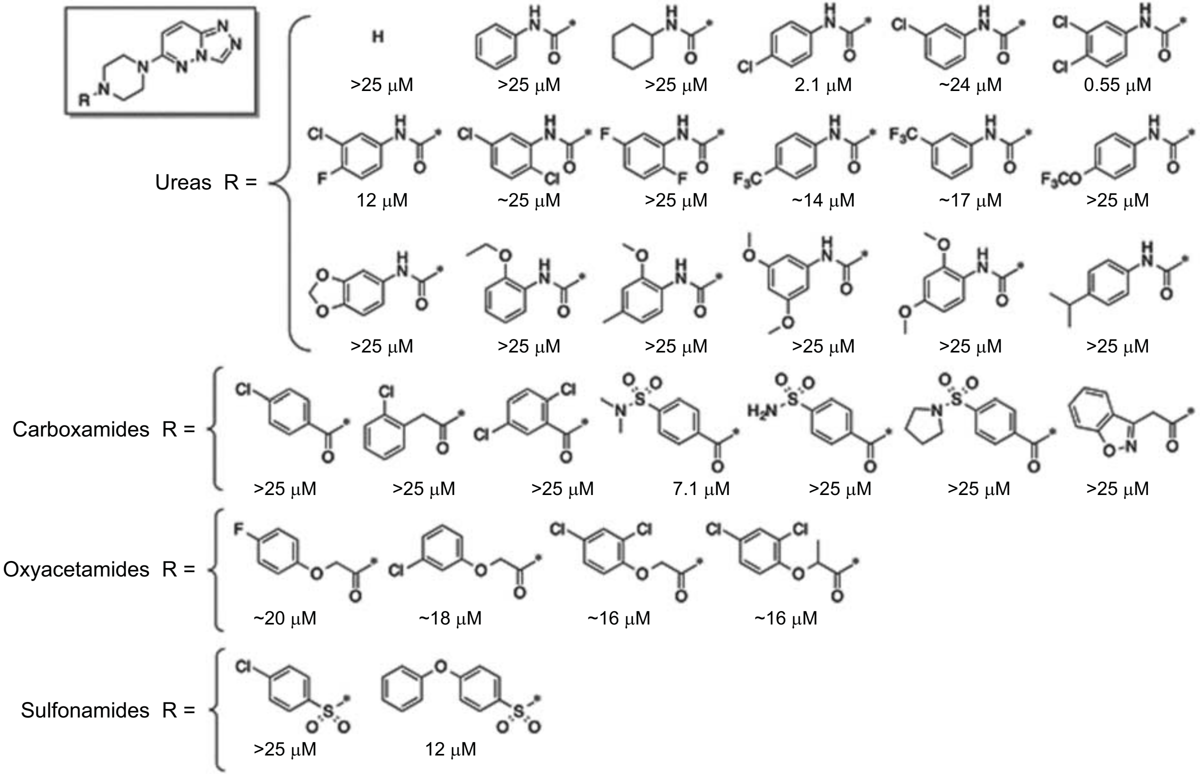

Supplementary Figure 2. Preliminary structure activity relationship studies using commercially available variants on the left-hand side R position. The mean EC_{50} for 2 biological replicates is shown, as measured against *C. parvum* in HCT-8 cells.

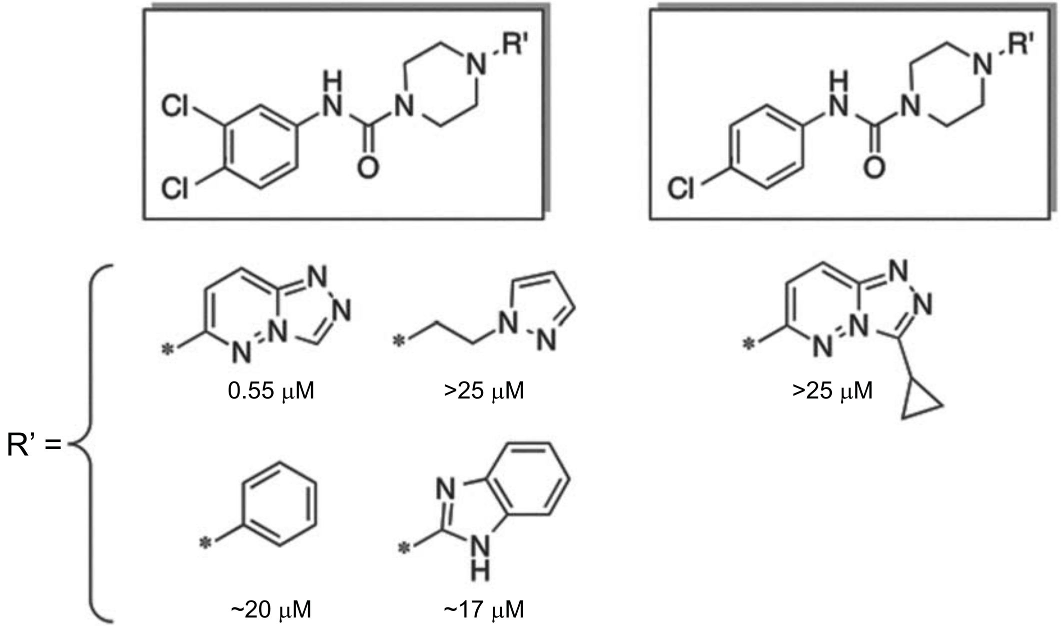

Supplementary Figure 3. Preliminary structure activity relationship studies using commercially available variants on the right-hand side R' position. The mean EC_{50} for 2 biological replicates is shown, as measured against *C. parvum* in HCT-8 cells.

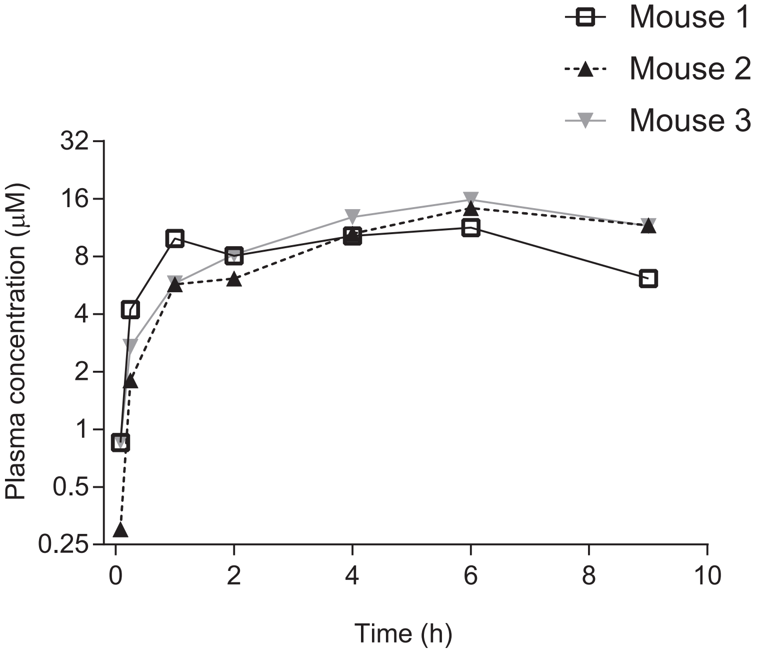

Supplementary Figure 4. Plasma pharmacokinetic information. Male CD-1 mice were administered 55 mg/kg of MMV665917 orally, after which plasma levels were measured at the indicated times (data for individual mice are shown).

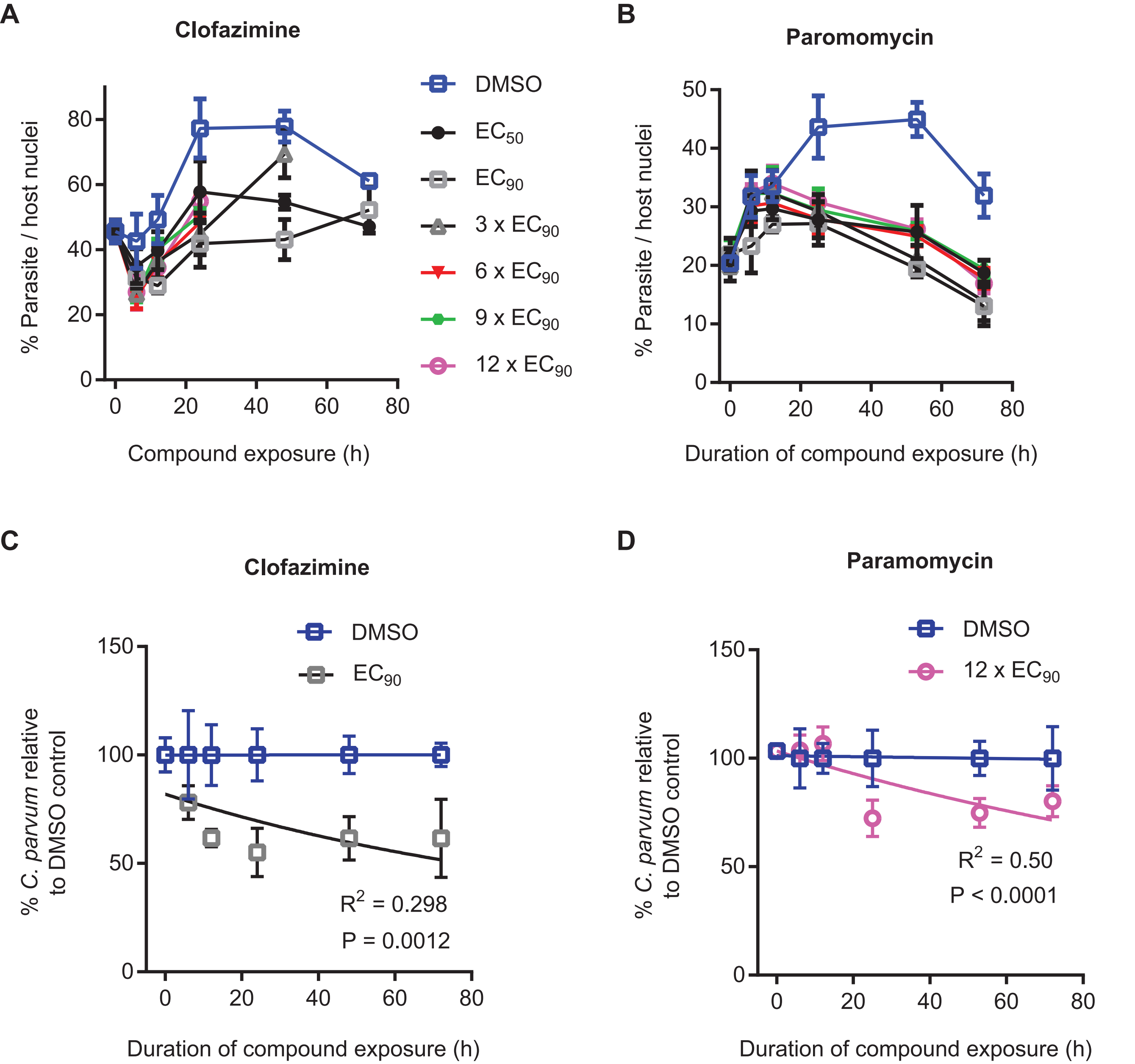

Supplementary Figure 5. Clofazimine and paromomycin both appear to be static for *C. parvum*. (A and B) Parasite persistence assay data for the highest non-toxic concentrations of (A) clofazimine and (B) paromomycin. (C and D) Parasite elimination curves for (C) clofazimine and (D) paromomycin. These data were normalized to the DMSO control for each time point; GraphPad Prism was used to try to fit a single-phase exponential decay curve. For both drugs, it is not possible to fit a decay curve (replicates test; $p < 0.05$). Data are representative of 3 independent experiments (mean and SD; $n = 4$ per experiment).

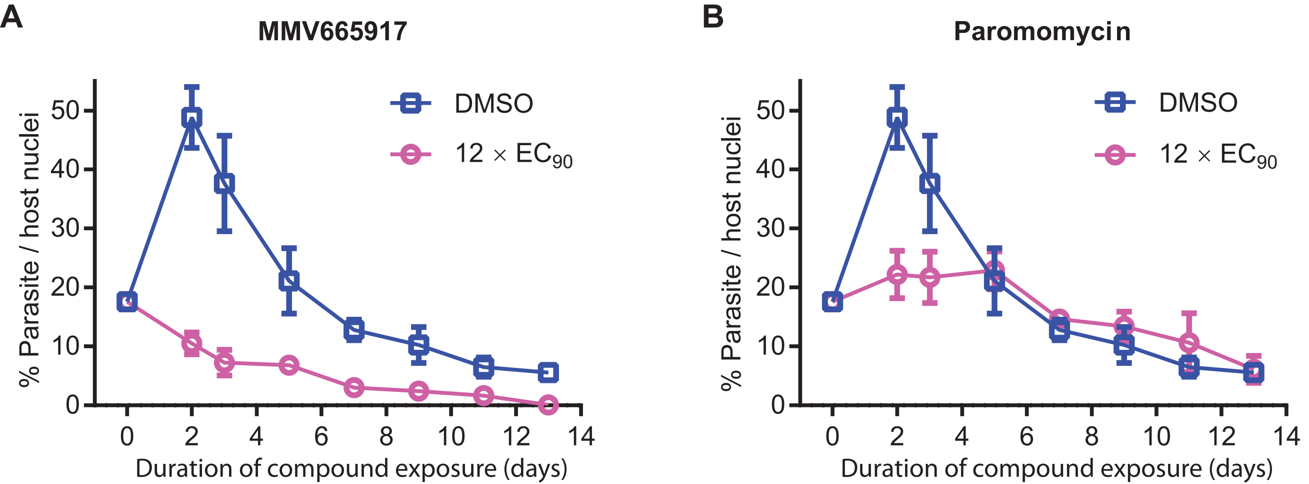

Supplementary Figure 6. Effects of prolonged MMV665917 vs. paromomycin exposure. The parasite persistence assay was extended by replacing the media and compound every 3 days for a total of 14 days of infection (i.e. 13 days of drug exposure). (A) MMV665917 treatment. (B) Paromomycin treatment. Data points are the mean and SD (n=12).

Table S1 Open access Malaria Box anti-*C. parvum* confirmed screening hits using new hit-definition

Compound ID	Smiles	Repurchased source	Mol wt (g/mol)	ALogP	*C. parvum* EC_{50} (95% CI) (μM)[b]
MMV006169[a]	C(Nc1nc(Nc2ccccc2)nc3ccccc13)c4ccccc4	Biomolecules	326.39	4.28	1.5 (1.2-1.9)
MMV403679[a]	c1(c(cnn1c2cccc(C)c2)C(=O)N3)N=C3n4nc(C)cc4NC(=O)c5cc(cccc6)c6o5	SPECS	465.46	4.34	0.12 (0.10-0.14)
MMV009085	OCCCCN1C(=O)c2ccc3C(=O)N(CCCCO)C(=O)c4ccc(C1=O)c2c34	SPECS	410.42	1.01	2.6 (1.9-3.5)
MMV665852	Clc1ccc(NC(=O)Nc2ccc(Cl)c(Cl)c2)cc1Cl	SPECS	350.03	5.18	3.4 (3.1-3.7)
MMV000720[a]	Cc1ccnc(NC(c2cccc(OCc3ccccc3)c2)c4ccc5cccnc5c4O)c1	ChemDiv	447.53	6.22	0.21 (0.17-0.27)
MMV001246[a]	CSc1ccccc1C(=O)Nc2nc(cs2)c3ccccn3	ChemDiv	327.42	3.38	1.8 (1.3-2.6)
MMV665814[a]	Oc1c(ccc2cccnc12)C(Nc3ccccn3)c4cccc(Oc5ccccc5)c4	SPECS	419.47	5.73	0.59 (0.52-0.67)
MMV665917	Clc1ccc(NC(=O)N2CCN(CC2)c3ccc4nncn4n3)cc1	SPECS	357.80	1.69	2.1 (1.9-2.3)
MMV665941[a]	CN(C)c1ccc(cc1)C(O)(c2ccc(cc2)N(C)C)c3ccc(cc3)N(C)C	ChemBridge	389.53	4.76	0.83 (0.69-1.0)
MMV666054[a]	COc1ccc(cc1)C(=O)NC(c2ccc(Cl)cc2Cl)c3cc(Cl)c4cccnc4c3O	ChemBridge	487.76	6.15	0.81 (0.58-1.1)
MMV006753	CC1=CC(=O)Oc2c1ccc3oc(C(=O)c4ccccc4)c(C)c23	SPECS	318.32	4.83	0.25 (0.03-2.3)
MMV011944	n1c(NCCO)c2c(cccc2)nc1Nc3cccc(OC)c3	SPECS	310.35	2.14	3.5 (2.9-4.3)
MMV665909	Brc1ccccc1C(=O)Nc2nc(cs2)c3ccccn3	SPECS	360.23	3.59	3.3 (2.9-3.8)
MMV000760	Oc1c(CN2CCN(CC2)c3ccccc3F)cc(Br)c4cccnc14	SPECS	416.29	4.43	0.29 (0.23-0.37)
MMV665969[a]	COc1cccc(c1)C(=O)NC(c2ccc(C)cc2)c3cc(Cl)c4cccnc4c3O	ChemBridge	432.90	5.31	0.054 (0.041-0.070)

[a] Previously repurchased and confirmed hits from reference 1

[b] EC_{50}, indicates 50% inhibitory concentration

Table S2 Summary of NSG mouse efficacy experiments for Malaria Box screening hits

Compound ID	Smiles	Supplier	Dose (mg/kg)	Interval (h)	Duration (days)	Vehicle	NSG efficacy
MMV006169 v1	C(Nc1nc(Nc2ccccc2)nc3ccccc13)c4ccccc4	Life Chemicals	50	12	4	1% HPMC[a] / 5% DMSO	No
MMV006169 v2	C1CCC(C1)Nc1nc(NCc2ccccc2)c2c(n1)cccc2	Life Chemicals	50	12	4	1% HPMC[a] / 5% DMSO	No
MMV006169 v3	COc1ccc(cc1Cl)Nc1nc(NCc2ccccc2Cl)c2c(n1)cccc2	Life Chemicals	50	12	4	1% HPMC[a] / 5% DMSO	No
MMV403679 v1	c31c(cnn1-c2cc(ccc2)C)C(=O)NC(=N3)n4c(cc(n4)C)NC(=O)c5c(cc(cc5)OC)OC	Life Chemicals	100	24	7	1% HPMC[a] / 5% DMSO	No
MMV403679 v2	c31c(cnn1-c2cc(ccc2)C)C(=O)NC(=N3)n4c(cc(n4)C)NC(=O)C5CC5	Life Chemicals	100	24	7	1% HPMC[a] / 5% DMSO	No
MMV403679 v3	c31c(cnn1-c2cc(ccc2)C)C(=O)NC(=N3)n4c(cc(n4)C)NC(=O)C5CCCC5	Life Chemicals	100	24	7	1% HPMC[a] / 5% DMSO	No
MMV403679 v4	c31c(cnn1-c2ccccc2)C(=O)NC(=N3)n4c(cc(n4)C)NC(=O)c5oc6c(c5)cccc6	Life Chemicals	100	24	7	1% HPMC[a] / 5% DMSO	No
MMV403679 v5	c31c(cnn1-c2cc(ccc2)C)C(=O)NC(=N3)n4c(cc(n4)C)N	Life Chemicals	100	24	7	1% HPMC[a] / 5% DMSO	No
MMV009085	OCCCCN1C(=O)c2ccc3C(=O)N(CCCCO)C(=O)c4ccc(C1=O)c2c34	-	-	-	-	-	ND
MMV665852	Clc1ccc(NC(=O)Nc2ccc(Cl)c(Cl)c2)cc1Cl	-	-	-	-	-	ND
MMV000720	Cc1ccnc(NC(c2cccc(OCc3ccccc3)c2)c4ccc5cccnc5c4O)c1	-	-	-	-	-	ND
MMV001246	CSc1ccccc1C(=O)Nc2nc(cs2)c3ccccn3	Enamine	50	12	4	1% HPMC[a] / 5% DMSO	No
MMV665814	Oc1c(ccc2cccnc12)C(Nc3ccccn3)c4cccc(Oc5ccccc5)c4	SPECS	50	12	4		No
MMV665917	Clc1ccc(NC(=O)N2CCN(CC2)c3ccc4nncn4n3)cc1	Life Chemicals	30	12	4	1% HPMC[a] / 5% DMSO	Yes
MMV665941	CN(C)c1ccc(cc1)C(O)(c2ccc(cc2)N(C)C)c3ccc(cc3)N(C)C	SPECS	50	12	4	1% HPMC[a] / 5% DMSO	No
MMV666054	COc1ccc(cc1)C(=O)NC(c2ccc(Cl)cc2Cl)c3cc(Cl)c4cccnc4c3O	-	-	-	-	-	ND
MMV006753	CC1=CC(=O)Oc2c1ccc3oc(C(=O)c4ccccc4)c(C)c23	-	-	-	-	-	ND
MMV011944	n1c(NCCO)c2c(cccc2)nc1Nc3cccc(OC)c3	-	-	-	-	-	ND
MMV665909	Brc1ccccc1C(=O)Nc2nc(cs2)c3ccccn3	Enamine	50	12	4	1% HPMC[a] / 5% DMSO	No
MMV000760	Oc1c(CN2CCN(CC2)c3ccccc3F)cc(Br)c4cccnc14	-	-	-	-	-	ND
MMV665969	COc1cccc(c1)C(=O)NC(c2ccc(C)cc2)c3cc(Cl)c4cccnc4c3O	SPECS	50	12	4	1% HPMC[a] / 5% DMSO	No

[a]HPMC, hydroxypropyl methyl cellulose

Table S3 Structure-activity relationship of MMV665917

Compound_ID	Supplier	SMILES	Mol wt (g/mol)	miLogP[a]	TPSA[b](Å^2)	*C. parvum* EC_{50} (95% CI) (µM)[c]
D-1 (MMV665917)	Life Chemicals	Clc1ccc(NC(=O)N2CCN(CC2)c3ccc4nncn4n3)cc1	357.81	2.40	78.67	2.1 (1.9-2.3)
D-2	Vitas M Labs	CN1CCN(CC1)c1ccc2n(n1)c(nn2)C(F)(F)F	286.26	1.37	49.57	>25
D-3	Vitas M Labs	FC(c1nnc2n1nc(cc2)N1CCN(CC1)Cc1ccccc1)(F)F	362.35	2.77	49.57	>25
D-4	Vitas M Labs	CCOC(=O)N1CCN(CC1)c1ccc2n(n1)c(nn2)C(F)(F)F	344.29	1.82	75.87	>25
D-9	Vitas M Labs	c1ccc(cc1)N1CCN(CC1)c1ccc2n(n1)cnn2	280.33	2.07	49.57	>25
D-12	Vitas M Labs	CC(=O)N1CCN(CC1)c1ccc2n(n1)c(nn2)C(F)(F)F	314.27	0.74	66.64	>25
D-18	Life Chemicals	O=C(N1CCN(CC1)c1ccc2n(n1)cnn2)Nc1ccccc1	323.35	1.72	78.67	>25
D-19	Life Chemicals	O=C(N1CCN(CC1)c1ccc2n(n1)cnn2)Nc1ccc2c(c1)OCO2	367.36	1.61	97.13	>25
D-20	Life Chemicals	O=C(N1CCN(CC1)c1ccc2n(n1)cnn2)NC1CCCCC1	329.40	2.48	78.67	>25
D-21	Life Chemicals	O=C(N1CCN(CC1)c1ccc2n(n1)c(C)nn2)NCc1ccccc1	351.41	2.06	78.67	>25
D-22	Life Chemicals	O=C(N1CCN(CC1)c1ccc2n(n1)c(nn2)C1CC1)NC1CCCCC1	369.46	2.94	78.67	>25
D-23	Life Chemicals	Clc1ccc(cc1)S(=O)(=O)N1CCN(CC1)c1ccc2n(n1)cnn2	378.84	1.96	83.71	>25
D-26	Life Chemicals	Fc1ccc(cc1)OCC(=O)N1CCN(CC1)c1ccc2n(n1)cnn2	356.35	1.54	75.87	20 (17-23)
D-27	Life Chemicals	O=C(N1CCN(CC1)c1ccc2n(n1)cnn2)Nc1cccc(c1)C(F)(F)F	391.35	2.59	78.67	17 (14-22)
D-28	Life Chemicals	O=C(N1CCN(CC1)c1ccc2n(n1)cnn2)Nc1ccc(c(c1)Cl)Cl	392.24	3.01	78.67	0.55 (0.46-0.65)
D-29	Life Chemicals	Fc1ccc(cc1)C(=O)N1CCN(CC1)c1ccc2n(n1)c(nn2)C1CC1	366.39	1.55	66.64	>25
D-31	Vitas M Labs	COc1ccc(cc1)N1CCN(CC1)c1ccc2n(n1)cnn2	310.35	2.13	58.80	>25
D-32	Life Chemicals	Clc1ccc(c(c1)NC(=O)N1CCN(CC1)c1ccc2n(n1)cnn2)Cl	392.24	3.01	78.67	~24.81
D-33	Life Chemicals	CCOc1ccccc1NC(=O)N1CCN(CC1)c1ccc2n(n1)cnn2	367.41	2.11	87.90	>25
D-34	Life Chemicals	COc1ccc(cc1NC(=O)N1CCN(CC1)c1ccc2n(n1)cnn2)C	367.41	2.16	87.90	>25
D-35	Life Chemicals	COc1cc(cc(c1)OC)NC(=O)N1CCN(CC1)c1ccc2n(n1)cnn2	383.40	1.76	97.13	>25
D-36	Life Chemicals	O=C(N1CCN(CC1)c1ccc2n(n1)cnn2)Nc1ccc(cc1)C(C)C	365.43	3.23	78.67	>25
D-37	Life Chemicals	Fc1ccc(c(c1)NC(=O)N1CCN(CC1)c1ccc2n(n1)cnn2)F	359.33	1.98	78.67	>25
D-38	Life Chemicals	O=C(N1CCN(CC1)c1ccc2n(n1)cnn2)Nc1ccc(c(c1)Cl)F	375.79	2.49	78.67	12 (11-14)
D-40	Life Chemicals	COc1ccc(cc1NC(=O)N1CCN(CC1)c1ccc2n(n1)cnn2)OC	383.40	1.76	97.13	>25
D-41	Life Chemicals	Clc1cccc(c1)OCC(=O)N1CCN(CC1)c1ccc2n(n1)cnn2	372.81	2.03	75.87	18 (16-21)
D-42	Life Chemicals	O=C(N1CCN(CC1)c1ccc2n(n1)cnn2)Cc1ccccc1Cl	356.81	2.13	66.64	>25
D-43	Life Chemicals	O=C(N1CCN(CC1)c1ccc2n(n1)c(nn2)C1CC1)Nc1ccc(c(c1)Cl)F	415.85	2.94	78.67	>25
D-44	Life Chemicals	O=C(c1ccc(cc1)S(=O)(=O)N(C)C)N1CCN(CC1)c1ccc2n(n1)cnn2	415.47	0.25	104.02	7.1 (5.9-8.5)
D-45	Life Chemicals	O=C(c1ccc(cc1)S(=O)(=O)N)N1CCN(CC1)c1ccc2n(n1)cnn2	387.42	-0.37	126.80	>25
D-46	Life Chemicals	Clc1ccc(cc1)C(=O)N1CCN(CC1)c1ccc2n(n1)cnn2	342.78	1.61	66.64	>25
D-47	Life Chemicals	Clc1ccc(c(c1)C(=O)N1CCN(CC1)c1ccc2n(n1)cnn2)Cl	377.23	2.22	66.64	>25
D-48	Life Chemicals	O=C(c1ccc(cc1)S(=O)(=O)N1CCCC1)N1CCN(CC1)c1ccc2n(n1)cnn2	441.51	0.65	104.02	>25
D-49	Life Chemicals	O=C(N1CCN(CC1)c1ccc2n(n1)cnn2)Cc1noc2c1cccc2	363.37	1.53	92.67	>25
D-50	Life Chemicals	Clc1ccc(c(c1)Cl)OCC(=O)N1CCN(CC1)c1ccc2n(n1)cnn2	407.25	2.66	75.87	16 (14-18)
D-51	Life Chemicals	Clc1ccc(c(c1)Cl)OC(C(=O)N1CCN(CC1)c1ccc2n(n1)cnn2)C	421.28	3.02	78.87	>25
D-53	Life Chemicals	N1CCN(CC1)c1ccc2n(n1)cnn2	204.23	-0.22	58.36	>25
D-60	Vitas M Labs	O=C(N1CCN(CC1)c1ccc2n(n1)c(C)nn2)OC(C)(C)C	318.37	1.50	75.87	>25
D-74	Sigma	O=C(Nc1ccc(Cl)c(Cl)c1)N2CCN(CC2)c3ccccc3	350.25	4.56	35.57	20 (12-32)
D-75	Life Chemicals	FC(F)(F)c1ccc(cc1)NC(=O)N2CCN(CC2)c3ccc4nncn4n3	391.36	2.62	78.67	11 (9.3-13)
D-76	Life Chemicals	FC(F)(F)Oc1ccc(cc1)NC(=O)N2CCN(CC2)c3ccc4nncn4n3	407.36	2.69	87.90	>25
D-77	Life Chemicals	Clc1cccc(c1)NC(=O)N2CCN(CC2)c3ccc4nncn4n3	357.81	2.38	78.67	~23.67
D-78	Life Chemicals	O=C(N1CCN(C2=NN3C(C=C2)=NN=C3)CC1)NC5=CC=C(Cl)C=C5	357.80	2.40	78.67	>25
D-79	Life Chemicals	O=S(N1CCN(C2=NN3C(C=C2)=NN=C3)CC1)(C4=CC=C(OC5=CC=CC=C5)C=C4)=O	436.50	3.04	92.94	12 (9.2-16)
D-80	Life Chemicals	O=C(N1CCN(C2=NC3=C(C=CC=C3)N2)CC1)NC4=CC=C(Cl)C(Cl)=C4	390.27	4.38	64.26	17 (14-20)
D-81	Life Chemicals	O=C(N1CCN(CCN2N=CC=C2)CC1)NC3=CC=C(Cl)C(Cl)=C3	368.27	2.94	53.40	>25

[a] and [b], calculated using molinspiration property engine versions v2013.09, v2014.11, and v2016.10

[c] EC_{50}, indicates half maximal effective concentration

Supplemental reference:

1. Bessoff K, Spangenberg T, Foderaro J, Jumani RS, Ward GE, Huston CD. 2014. Identification of *Cryptosporidium parvum* active chemical series by repurposing the Open Access Malaria Box. Antimicrob Agents Chemother doi:10.1128/AAC.02641-13.
